# Priority research questions in atopic dermatitis: an International Eczema Council eDelphi consensus

**DOI:** 10.1111/bjd.19874

**Published:** 2021-04-07

**Authors:** K. Abuabara, S.G. Nicholls, S.M. Langan, E. Guttman‐Yassky, N.J. Reynolds, A.S. Paller, S.J. Brown, the International Eczema Council Priority Research Group

**Affiliations:** ^1^ Department of Dermatology University of California San Francisco (UCSF) San Francisco CA USA; ^2^ Ottawa Hospital Research Institute Ottawa Canada; ^3^ London School of Hygiene and Tropical Medicine London UK; ^4^ Icahn School of Medicine at Mount Sinai New York NY USA; ^5^ Newcastle University Newcastle upon Tyne UK; ^6^ Royal Victoria Infirmary Newcastle upon Tyne UK; ^7^ Northwestern University Feinberg School of Medicine Chicago IL USA; ^8^ University of Dundee Dundee UK; ^9^ Ninewells Hospital and Medical School Dundee UK

dear editor, Recent advances in understanding the complex pathogenesis of atopic dermatitis (AD, also known as eczema or atopic eczema), coupled with the development of new treatments, have led to increased interest from multiple stakeholders. There is a need to prioritize areas for research to inform a coordinated approach to advancing science and patient care. We sought to fill a gap in the literature, specifically from the perspective of clinicians involved in AD patient care and research.

Our objective was to identify and reach consensus on a set of research questions to be prioritized for future work in AD. We conducted a three‐round electronic Delphi (eDelphi) process with members of the International Eczema Council (IEC).^1^, ^2^ The IEC is a global nonprofit organization that aims to promote the optimal management of AD through research, education and patient/family care.

In the first round, participants provided online consent and submitted up to three research questions they believed were the highest priority in AD. These could include areas of uncertainty (i.e. questions that are not adequately answered by existing evidence) and/or unmet needs (i.e. areas where there is not currently ongoing or adequate research). Participants were asked to align each question to one of the following five domains: (i) epidemiology, including phenotype, disease course, disease/psychological burden and comorbidities; (ii) pathophysiology and molecular mechanisms, including genomics and immunology; (iii) translational research, including stratified/personalized/precision and systems medicine (including models); (iv) therapeutics, including nonpharmacological interventions such as psychological support and educational programmes; and (v) other. These domains were based on a pilot exercise to determine research priorities, carried out with IEC members in 2015, and previous systematic reviews in dermatology.^3^ Data were collected using REDCap software, and free‐text responses were reviewed independently by two researchers.^4^ Duplicate and overlapping submissions were aggregated through discussion with the investigator team.

Round 1 was completed by 68 of 82 invited participants (83%). Respondents were from 22 countries; 96% were physicians and 90% were based at teaching hospitals. Among those caring for patients with AD, 45% cared primarily for adults, 22% primarily for children and 33% for both. After consolidation, 62 of 197 priority research questions were put forward to round 2.

In the second and third rounds, participants were asked to score each of the submitted questions on a scale from one to nine using the COMET Initiative Delphi Manager software.^5^ Consensus was predefined as > 70% of participants scoring the importance of an item as seven to nine (critically important) and < 15% of participants scoring it as one to three (not important). Questions that did not meet these criteria after round 2 were dropped, and in round 3, participants were shown the groups’ scores and asked to re‐score each of the remaining questions.

After the final round, eight research questions achieved consensus and are listed in Table 1. These spanned all domains and focused on: prediction of disease course; identification of disease subtypes; evaluation of safe, effective and disease‐modifying therapies; comparative effectiveness of treatments; biomarker assessment; and mechanisms and treatment of disease flares. The consensus that identification of subtypes remains a priority area for further research is consistent with recent work in the UK, in which the need to identify subtypes of patients with differing treatment responses was identified as a clear priority.^6^

**Table 1:** Priority research questions that met consensus criteria

Priority research question (domain)^a^	% scored, 1–3 ‘not important’	% scored, 7–9 ‘critically important’	% scored, 1–3 ‘not important’	% scored, 7–9 ‘critically important’
Round 1: submission of questions, 82 IEC members invited, 68 respondents, 197 questions submitted; consolidated to 62 priority questions for voting	Round 2: voting, 93 IEC members invited,^b^ 63 respondents, 8 questions met criteria		Round 3: repeat voting, 63 IEC members invited, 59 respondents, 8 questions again met criteria	
Can we predict who will develop chronic disease; associated comorbidities and/or adverse outcomes? (epidemiology)	3	76	0	83
Can clinically meaningful subtypes of AD be defined based on age at onset; genetics; environmental factors; and clinical features? (epidemiology)	5	71	0	82
How do we best classify AD (disease endotype) to predict clinical outcomes (e.g. prognosis; systemic disease) and therapeutic outcomes (drug endotype)? (pathophysiology)	0	73	0	88
Which therapeutic strategies can prevent/modify the course of AD and prevent the development of comorbidities? (therapeutics, epidemiology, translational)	0	90	0	88
Which topical and systemic treatments are safest and most effective for short‐ and long‐term disease control? (therapeutics)	3	77	2	85
What is the comparative effectiveness and side‐effect profile of systemic AD treatments (both classical and new)? (therapeutics)	3	72	0	97
How can AD be subclassified using biomarker assessments and other tests in ways that allow better prediction of severity; disease course; treatment response; and comorbidities? (translational, pathophysiology, therapeutics)	5	78	2	85
What are the mechanisms and potential therapeutic strategies to reduce and control disease flares in AD? (translational)	0	70	0	85

AD, atopic dermatitis; IEC, International Eczema Council; ^a^Similar research questions submitted under more than one domain were combined after round 1 and listed with multiple domains; ^b^Additional participants who had become Councillors or Associates of the IEC after the completion of round 1 were invited to participate in round 2, increasing the total group number.

Our objective was to fill a gap in the literature on research priorities from the academic clinician/researcher perspective, given that prior efforts have examined patient, translational and economic research priorities.^6^, ^7^, ^8^ The research questions identified reveal a different perspective from some patient‐led priority‐setting exercises, in which the need for research into practical issues, such as use of topical steroids and emollients and food allergy testing, were highlighted.^8^

Strengths of our work include high response rates and the clear consensus that emerged. Limitations relate to its generalizability and the extent to which the priority research questions reflect all stakeholder priorities for AD research. Respondents were directly involved in patient care and reported expertise in various types of AD research but were predominantly from university teaching hospitals. Geographically, they worked in six continents with differing socioeconomic contexts, but North America and Europe were overrepresented. This eDelphi exercise was completed in February 2020 and thus does not reflect changing priorities as a result of the global COVID‐19 pandemic.

The research questions prioritized indicate the need for multidisciplinary research including epidemiology, clinical trials and molecular medicine to address the outstanding challenges in understanding this complex disease and optimizing patient care.

## Author Contribution

**Katrina Abuabara:** Conceptualization (equal); Data curation (equal); Formal analysis (equal); Investigation (equal); Methodology (equal); Project administration (equal); Supervision (lead); Writing‐original draft (lead); Writing‐review & editing (equal). **Stuart Nicholls:** Conceptualization (supporting); Data curation (supporting); Investigation (supporting); Methodology (supporting); Project administration (supporting); Writing‐review & editing (supporting). **Sinead Langan:** Conceptualization (supporting); Data curation (supporting); Formal analysis (supporting); Investigation (supporting); Methodology (supporting); Project administration (supporting); Writing‐review & editing (supporting). **Emma Guttman‐Yassky:** Conceptualization (supporting); Data curation (supporting); Formal analysis (supporting); Investigation (supporting); Methodology (supporting); Project administration (supporting); Writing‐review & editing (supporting). **Nick Reynolds:** Conceptualization (supporting); Data curation (supporting); Investigation (supporting); Methodology (supporting); Project administration (supporting); Writing‐review & editing (supporting). **Amy Paller:** Conceptualization (supporting); Data curation (supporting); Formal analysis (supporting); Investigation (supporting); Methodology (supporting); Project administration (supporting); Writing‐review & editing (supporting). **Sara Brown:** Conceptualization (equal); Data curation (equal); Formal analysis (equal); Investigation (equal); Methodology (equal); Project administration (equal); Writing‐original draft (supporting); Writing‐review & editing (supporting).

## Supporting information

**Appendix S1** Funding and Conflicts of interest statements.
**Appendix S2** Affiliations for the International Eczema Council Priority Research Group.

## The International Eczema Council Priority Research Group

Affiliations for the members of the International Eczema Council Priority Research Group may be found in Appendix S2 in the Supporting Information.

Tove Agner, Valeria Aoki, Martine Bagot, Sebastien Barbarot, Lisa Beck, Thomas Bieber, Robert Bissonnette, Andrew Blauvelt, Patrick M. Brunner, David E. Cohen, Michael J. Cork, Anna De Benedetto, Mette Deleuran, Sandipan Dhar, Ncoza Dlova, Aaron M. Drucker, Lawrence Eichenfield, James T. Elder, Kilian Eyerich, Carsten Flohr, Carlo Gelmetti, Giampiero Girolomoni, Melinda J. Gooderham, Emma Guttman, Jon M. Hanifin, DirkJan Hijnen, Emmilia Hodak, Alan D Irvine, Kenji Kabashima, Norito Katoh, Kyu Han Kim, Heidi Kong, Cheng Che E Lan, Kwang Hoon Lee, Yael Anne Leshem, Danielle Marcoux, Uffe Nygaard, Chang Ook Park, Carle Paul, Marieke Seyger, Elaine Siegfried, Jonathan Silverberg, Eric Simpson, Jean Francois Stalder, Sonja Stander, Martin Steinhoff, John Su, Jacek Szepietowski, Roberto Takaoka, Jacob P. Thyssen, Christian Vestergaard, Miriam Weinstein, Yik Weng Yew.
