# Supplementary material for: Priority research questions in atopic dermatitis: an International Eczema Council eDelphi consensus
Source: Br J Dermatol. 2021 Apr 7;185(1):203–5. doi: 10.1111/bjd.19874 (PMC8359998; doi:10.1111/bjd.19874)
Supplement: Supplementary file 1 — Appendix S1 Funding and Conflicts of interest statements. Appendix S2 Affiliations for the International Eczema Council Priority Research Group. [file BJD-185-203-s001.docx]

**Appendix S1** Funding and Conflicts of interest statements.

**Funding:** This project was not tied to specific funding and authors and collaborators did not receive any direct compensation for their participation. K.A. receives funding from the National Institute of Arthritis and Musculoskeletal and Skin Diseases (NIAMS, K23AR073915). S.J.B. and S.M.L. hold Senior Research Fellowships awarded by the Wellcome Trust (ref 106865/Z/15/Z) and (205039/Z/16/Z), respectively. N.J.R.’s research/laboratory is funded in part by the Newcastle NIHR Biomedical Research Centre, and the Newcastle NIHR Medtech and In vitro Diagnostic Co-operative, and he is a National Institute for Health Research (NIHR) Senior Investigator. The International Eczema Council is a global nonprofit organization that receives unrestricted grants from corporate sponsors; corporate sponsors and industry liaisons were not involved in this research. The opinions expressed in this article do not reflect the view of the funding bodies.

**Conflicts of interest statement:** K.A. is a consultant for TARGET Real World Evidence and has received research funds (grants paid to her institution) from Pfizer. S.G.N. has no conflicts to declare. S.M.L. holds research grant funding from the Wellcome Trust (ref 205039/Z/16/Z), the British Skin Foundation, and an EU IMI award ‘BIOMAP’ (Biomarkers in AD and Psoriasis). N.J.R. has received, through Newcastle University, research grant funding, funding for lectures and/or travel support from Celgene, Genentech and Sanofi-Genzyme. S.J.B. holds research grant funding from the Wellcome Trust (ref 106865/Z/15/Z), the British Skin Foundation, Tayside Dermatological Research Charity, Pfizer Investigator-Initiated Research and an EU IMI award ‘BIOMAP’ (Biomarkers in AD and Psoriasis). A.S.P. has been an investigator for AbbVie, Eli Lilly, Galderma, Incyte, Leo, Novartis, and Regeneron; Consultant with honorarium for Dermavant, Dermira, Eli Lilly, Forte, Galderma, Leo, Novartis, Pfizer, Regeneron and Sanofi-Genzyme. E.G.-Y. is an employee of Mount Sinai and has received research funds (grants paid to the institution) from AbbVie, Almirall, Amgen, AnaptysBio, Asana Biosciences, Boerhinger-Ingelhiem, Celgene, Dermavant, DS Biopharma, Eli Lilly, Galderma, Ichnos Sciences, Innovaderm, Janssen, Kiniska, Kyowa Kirin, Leo Pharma, Novan, Pfizer, Ralexar, Regeneron Pharmaceuticals, Inc., Sienna Biopharma, UCB and Union Therapeutics; and is a consultant for AbbVie, Aditum Bio, Almirall, Amgen, Asana Biosciences, AstraZeneca, Boerhinger-Ingelhiem, Cara Therapeutics, Celgene, Concert, DBV, Dermira, DS Biopharma, Eli Lilly, EMD Serono, Escalier, Galderma, Ichnos Sciences, Incyte Kyowa Kirin, Leo Pharma, Mitsubishi Tanabe, Pandion Therapeutics, Pfizer, RAPT Therapeutics, Regeneron Pharmaceuticals, Inc., Sanofi, Sienna Biopharma, Target Real World Evidence and Union Therapeutics.

**Appendix S2** Affiliations of the International Eczema Council Priority Research Group members.

Tove Agner, MD, DMSc, Bispebjerg Hospital, University of Copenhagen, Copenhagen, Denmark;

Valeria Aoki, MD, Department of Dermatology, Faculdade de Medicina, Universidade de São Paulo, São Paulo, Brazil;

Martine Bagot, MD, PhD, Department of Dermatology and Inserm U976 chez Hôpital Saint Louis, Paris, France;

Sebastien Barbarot, MD, PhD, MSc, Department of Dermatology, Nantes University Hospital, Nantes, France;

Lisa Beck, MD, Department of Dermatology, Medicine and Pathology, University of Rochester Medical Center, Rochester, NY, USA;

Thomas Bieber, MD, PhD, MDRA, Department of Dermatology and Allergology, Christine Kühne Center for Allergy Research and Education, Universität of Bonn, Bonn, Germany.;

Robert Bissonnette, MD, FRCPC, MSc, Innovaderm, Montreal, QC, Canada;

Andrew Blauvelt, MD, MBA, Oregon Medical Research Center, Portland, OR, USA;

Patrick M. Brunner, MD, MSc, Department of Dermatology, Medical University of Vienna, Vienna, Austria;

David E. Cohen, MD, MPH, Allergic, Occupational, and Environmental Dermatology, Ronald O Perelman Department of Dermatology, New York University School of Medicine, New York, NY, USA;

Michael J Cork, MD, PhD, FRCP, Department of Infection, Immunity & Cardiovascular Disease, University of Sheffield Medical School, Sheffield, UK;

Anna De Benedetto, MD, Department of Dermatology, College of Medicine, University of Florida, Gainesville, FL, USA;

Mette Deleuran, MD, DMSc, Department of Dermato-Venereology, Aarhus University Hospital, Aarhus N, Denmark;

Sandipan Dhar, MD, Department of Pediatric Dermatology, Institute of Child Health, Kolkata, India;

Ncoza Dlova, MBChB, FCDerm, PhD, Nelson R Mandela School of Clinical Medicine, University of KwaZulu Natal (UKZN), Durban, South Africa;

Aaron M Drucker, MD, ScM, FRCPC, Division of Dermatology, Department of Medicine, University of Toronto, Toronto, Ontario, Canada;

Lawrence Eichenfield, MD, Departments of Dermatology and Pediatrics, University of California, San Diego, California, Rady Children's Hospital, San Diego, CA, USA;

James T. Elder, MD, PhD, Molecular Genetic Dermatology, University of Michigan Medical School, Ann Arbor, MI, USA;

Kilian Eyerich, MD, PhD, Department of Dermatology and Allergy, Technical University Munich, Munich, Germany; the Center of Allergy and Environment (ZAUM), HMGU & Technical University of Munich;

Carsten Flohr, MD, PhD, MSc, Unit for Population-Based Dermatology Research, St John's Institute of Dermatology, Guy's and St Thomas’ NHS Foundation Trust and King's College, London;

Carlo Gelmetti, MD, PhD, Department of Pathophysiology and Transplantation, University of Milan, Head, Unit of Pediatric Dermatology, ""Ospedale Maggiore Policlinico"", Milan, Italy; "

Giampiero Girolomoni, MD, Section of Dermatology and Venereology, Department of Medicine, University of Verona, Verona, Italy;

Melinda J Gooderham, MD, Skin Centre for Dermatology, Peterborough, Ontario, Canada; Probity Medical Research, Waterloo, Ontario, Canada; Queen's University, Kingston, Ontario, Canada;

Emma Guttman, MD, PhD, Department of Dermatology, Icahn School of Medicine at Mount Sinai, New-York, NY, USA;

Jon M. Hanifin, MD, Department of Dermatology, Oregon Health & Science University, Portland, OR, USA;

DirkJan Hijnen, MD, PhD, the Dept of Dermatology, Erasmus University Medical Center (Erasmus MC), Rotterdam;

Emmilia Hodak, Department of Dermatology, Rabin Medical Center, Beilinson Hospital, Petah Tikva, Israel; Sackler Faculty of Medicine, Tel Aviv University, Tel Aviv, Israel;

Alan D Irvine, MD, DSc, Trinity College Dublin, Children’s Health Ireland, Dublin, Ireland;

Kenji Kabashima, MD, PhD, Dept of Dermatology, Kyoto University Graduate School of Medicine, Kyoto, Japan;

Norito Katoh, MD, PhD, Department of Dermatology, Kyoto Prefectural University of Medicine, Kyoto, Japan;

Kyu Han Kim, MD, PhD, Institute of Human-Environment Interface Biology, Seoul National University, College of Medicine, Department of Dermatology, Seoul Korea;

Heidi Kong, MD, MHSc, Cutaneous Microbiome and Inflammation Section, Dermatology Branch, NIAMS, NIH, Bethesda, MD 20892, USA;

Cheng Che E Lan, MD, PhD, Department of Dermatology, Kaohsiung Medical University Hospital, Kaohsiung 80708, Taiwan;

Kwang Hoon Lee, MD, PhD, Dermatology and Cutaneous Biology Research Institute, Yonsei University College of Medicine, Seoul, Korea;

Yael Anne Leshem, MD, MCR, Division of Dermatology, Rabin Medical Center-Beilinson Hospital, Petah Tikva, Israel, Sackler Faculty of Medicine, Tel Aviv University, Tel Aviv, Israel;

Danielle Marcoux, MD, Department of Pediatrics, Division of Dermatology, University of Montreal and CHU Sainte-Justine, Montreal, QC, Canada;

Uffe Nygaard, MD, PhD, Aarhus University Hospital, Department of Dermatology, Denmark;

Chang Ook Park, MD, PhD, Severance Hospital, Department of Dermatology Yonsei University College of Medicine, South Korea;

Carle Paul, MD, PhD, Department of Dermatology Allergology, Toulouse University and CHU, Toulouse, France;

Marieke Seyger, MD, PhD, Department of Dermatology, Radboud University Medical Centre, Nijmegen, The Netherlands.;

Elaine Siegfried, MD, Professor of Pediatrics and Dermatology; Saint Louis University School of Medicine, St. Louis, MO USA;

Jonathan Silverberg, MD, PhD, MPH, The George Washington University School of Medicine and Health Sciences, Washington, DC, USA;

Eric Simpson, MD, MCR, Dermatology, Oregon Health & Science University School of Medicine, Portland, OR, USA;

Jean Francois Stalder, MD, Department of Dermatology, Nantes University Hospital, Nantes, France;

Sonja Stander, MD, Center for Chronic Pruritus, Dept of Dermatology, Chief Physician, Münster University Hospital, Munster, Germany;

Martin Steinhoff, MD, PhD, MSc, FRCPI, Department of Dermatology and Venereology, Weill Cornell MedicineQatar, Hamad Medical Corporation, Doha, Qatar;

John Su, MD, MEpi, MA, MSt, Departments of Dermatology and Pediatrics, Murdoch Children's Research Institute, University of Melbourne and Monash University, Eastern Health, Melbourne, VIC, Australia;

Jacek Szepietowski, MD, PhD, Dept of Dermatology, Venereology and Allergology, Wroclaw Medical University, Wroclaw, Poland;

Roberto Takaoka, MD, Department of Dermatology, University of São Paulo Medical School, São Paulo, Brazil;

Jacob P. Thyssen, PhD, DmSci, Department of Dermatology and Allergy, Herlev-Gentofte Hospital, Univ of Copenhagen, Hellerup, Denmark;

Christian Vestergaard, MD, PhD, MSDc, Department of Dermatology, Aarhus University Hospital, Aarhus, Denmark;

Miriam Weinstein, BSc; B.Sc.N; MD FRCPC (Paediatrics and Dermatology), Section of Dermatology, Division of Paediatric Medicine, The Hospital for Sick Children, Toronto, Canada;

Yik Weng Yew, MBBS, MPH, National Skin Centre, Singapore
